# Supplementary material for: FEVER: an interactive web-based resource for evolutionary transcriptomics across fishes
Source: Nucleic Acids Res. 2024 Apr 8;52(W1):W65–9. doi: 10.1093/nar/gkae264 (PMC11223851; doi:10.1093/nar/gkae264)
Supplement: gkae264_Supplemental_File [file gkae264_supplemental_file.pdf]

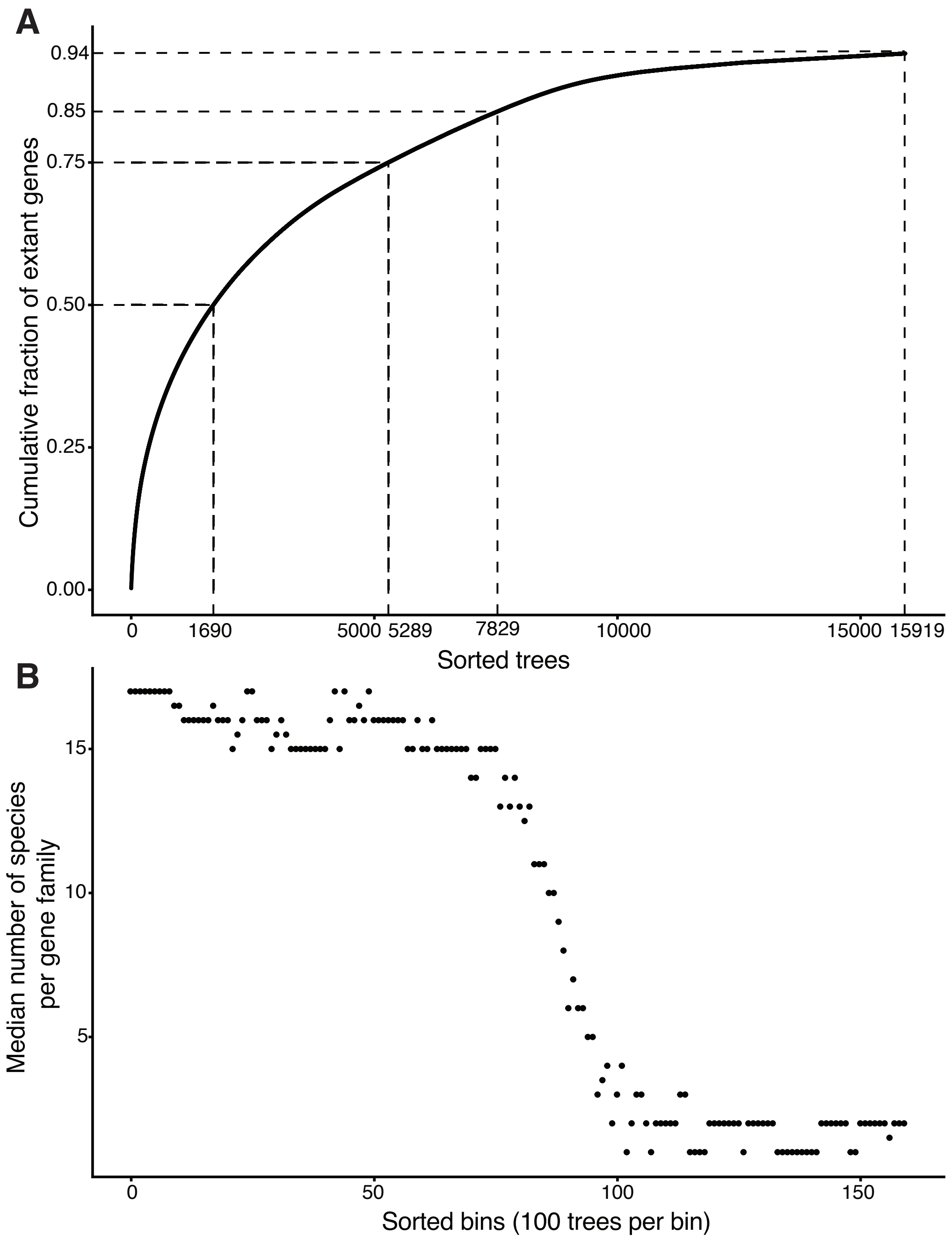

Supplementary Figure 1: Characterization of gene families. (A) Cumulative fraction of all extant genes covered by gene families. Gene families are sorted decreasingly based on its number of genes on the x axis. (B) Median number of species per gene family. Gene families are sorted and binned (100 trees per bin) decreasingly based on its number of genes on the x axis.

species scientific name	species common name	assembly	ncbi	Ensembl	#genes	#genes in trees	Fraction of genes in trees
Oncorhynchus mykiss	rainbow trout	USDA_OmykA_1.1	GCF_013265735.2	NA	41123	40326	0.980619118
Salmo trutta	brown trout	fSalTru1.1	GCF_901001165.1	NA	42094	41149	0.977550245
Umbra pygmaea	eastern mudminnow	ASM1680114v1	GCA_016801145.1	NA	24516	23102	0.942323381
Esox lucius	northern pike	Eluc_V3	GCA_000721915.3	ensembl V95	23875	23326	0.977005236
Oryzias latipes	medaka	ASM223467v1	GCA_002234675.1	ensembl V95	23560	22658	0.961714771
Gadus morhua	atlantic cod	gadMor1	NA	ensembl V95	20093	19290	0.960035833
Astyanax mexicanus	mexican tetra	Astyanax_mexicanus-2.0	GCA_000372685.2	ensembl V95	26584	25373	0.954446283
Pangasianodon hypophthalmus	striped catfish	GENO_Phyp_1.0	GCF_009078355.1	NA	22742	22441	0.986764577
Danio rerio	zebrafish	GRCz11	GCA_000002035.4	ensembl V95	24909	24200	0.971536392
Alosa alosa	allis shad	AALO_Geno_1.1	GCA_017589495_2	NA	26440	24082	0.910816944
Anguilla anguilla	european eel	fAngAng1.pri	GCF_013347855.1	NA	25888	25492	0.984703337
Lepisosteus oculatus	spotted gar	LepOcu1	GCA_000242695.1	ensembl V95	18335	18059	0.984946823
Amia calva	bowfin	AmiCal1	GCA_017591415.1	NA	21948	20360	0.927647166
Mus musculus	mouse	GRCm38.p6	GCA_000001635.8	ensembl V95	22040	21377	0.96991833
Homo sapiens	human	GRCh38.p12	GCA_000001405.27	ensembl V95	19938	19495	0.977781121
Drosophila melanogaster	fruitfly	BDGP6	GCA_000001215.4	ensembl V95	13903	9594	0.690066892
Caenorhabditis elegans	roundworm	WBcel235	GCA_000002985.3	ensembl V95	20222	13323	0.65883691

Supplementary Table S1: Species and genomes used in this study
